# Sulfasalazine impacts on ferroptotic cell death and alleviates the tumor microenvironment and glioma-induced brain edema

**DOI:** 10.18632/oncotarget.8651

**Published:** 2016-04-08

**Authors:** Tina Sehm, Zheng Fan, Ali Ghoochani, Manfred Rauh, Tobias Engelhorn, Georgia Minakaki, Arnd Dörfler, Jochen Klucken, Michael Buchfelder, Ilker Y. Eyüpoglu, Nicolai Savaskan

**Affiliations:** ^1^ Translational Cell Biology & Neurooncology Laboratory, Department of Neurosurgery Schwabachanlage 6 (Kopfklinik), Universitätsklinikum Erlangen (UKER), Medical School of The Friedrich Alexander University of Erlangen-Nürnberg (FAU), 91054 Erlangen, Germany; ^2^ Department of Pediatrics and Adolescent Medicine, Universitätsklinikum Erlangen (UKER), Medical School of The Friedrich Alexander University of Erlangen-Nürnberg (FAU), 91054, Erlangen, Germany; ^3^ Department of Neuroradiology, Schwabachanlage 6 (Kopfklinik), Universitätsklinikum Erlangen (UKER), Medical School of The Friedrich Alexander University of Erlangen-Nürnberg (FAU), 91054, Erlangen, Germany; ^4^ Department of Molecular Neurology, Universitätsklinikum Erlangen(UKER), Medical School of The Friedrich Alexander University of Erlangen-Nürnberg (FAU), 91054 Erlangen, Germany; ^5^ BiMECON Ent., Berlin, Germany

**Keywords:** glioblastoma, cancer, tumor microenvironment, brain swelling

## Abstract

The glutamate transporter xCT (SCL7a11, system Xc-, SXC) is an emerging key player in glutamate/cysteine/glutathione homeostasis in the brain and in cancer. xCT expression correlates with the grade of malignancy. Here, we report on the use of the U.S. Food and Drug Administration and EMA-approved xCT inhibitor, sulfasalazine (SAS) in gliomas. SAS does not affect cell viability in gliomas at concentrations below 200 μM. At higher concentrations SAS becomes gliomatoxic. Mechanistically SAS inhibits xCT and induces ferroptotic cell death in glioma cells. There is no evidence for impact on autophagic flux following SAS application. However, SAS can potentiate the efficacy of the standard chemotherapeutic and autophagy-inducing agent temozolomide (Temcat, Temodal or Temodar®). We also investigated SAS in non-transformed cellular constituents of the brain. Neurons and brain tissue are almost non-responding to SAS whereas isolated astrocytes are less sensitive towards SAS toxicity compared to gliomas. *In vivo* SAS treatment does not affect experimental tumor growth and treated animals revealed comparable tumor volume as untreated controls. However, SAS treatment resulted in reduced glioma-derived edema and, hence, total tumor volume burden as revealed by T2-weighted magnetic resonance imaging. Altogether, we show that SAS can be utilized for targeting the glutamate antiporter xCT activity as a tumor microenvironment-normalizing drug, while crucial cytotoxic effects in brain tumors are minor.

## INTRODUCTION

High grade gliomas or malignant primary brain tumors (WHO III and IV) are the most common primary tumors in the central nervous system derived from glial cells or neuronal and glial progenitors, with glioblastomas being the most malignant entity [1], [2]. Despite multimodal therapy regimens including neurosurgical resection, radiotherapy and cytotoxic chemotherapy, neurooncologists still face a poor prognosis for glioma patients, i.e. less than 30% of patients survive more than two years [3]. Rapid proliferation, diffuse brain invasion as well as tumor-induced brain edema and glioma-induced brain damage are pathological hallmarks of these tumors and are likely to determine unfavorable prognosis [4]. Sulfasalazine (SAS) was developed some 80 years ago from a sulfapyridine moiety linked to 5-aminosalicylic acid (5-ASA) as an anti-inflammatory drug. Since then, SAS (brand name Azulfidine in the U.S., Salazopyrin in Europe) has been approved by the EU EMA and U.S. Food and Drug Administration and applied in clinical trials for rheumatic polyarthritis and in particular for chronic ulcerative colitis [5]. Due to colonic bacterial azoreductase activity, orally applied SAS gets metabolized and split into sulfapyridine and 5-aminosalocyl acid (5-ASA) in colon and acts there locally. In fact, 5-ASA has been shown to be the therapeutic active moiety of SAS in ulcerative colitis, and further investigations revealed a pleiotropic picture of action. Hence, sulfasalazine can scavenge reactive oxygen species [6], [7], inhibit leukocyte motility and IL-1 and IL-2 production [8], induce cancer apoptosis [9], and inhibit nuclear factor kappa B (NFκB) [10], [11]. Recent studies indicated that SAS can inhibit the glutamate antiporter xCT [12]. Moreover, SAS has been used *in vivo* and displayed alleviation of glioma-induced seizure activities [13], [14]. Here, we investigated in detail SAS on gliomas and its underlying cell death mechanisms *in vitro* and *in vivo*. Hence, we tested whether SAS can be included into a multimodal therapeutic approach in combination with the standard treatment for malignant gliomas. We have found that although SAS does not show crucial cytotoxic and chemotherapeutic effects, SAS impacts on gliomas as a tumor microenvironment-normalizing drug.

## RESULTS

### Sulfasalazine reduces glioma cell growth at high concentrations

**Figure 1:**
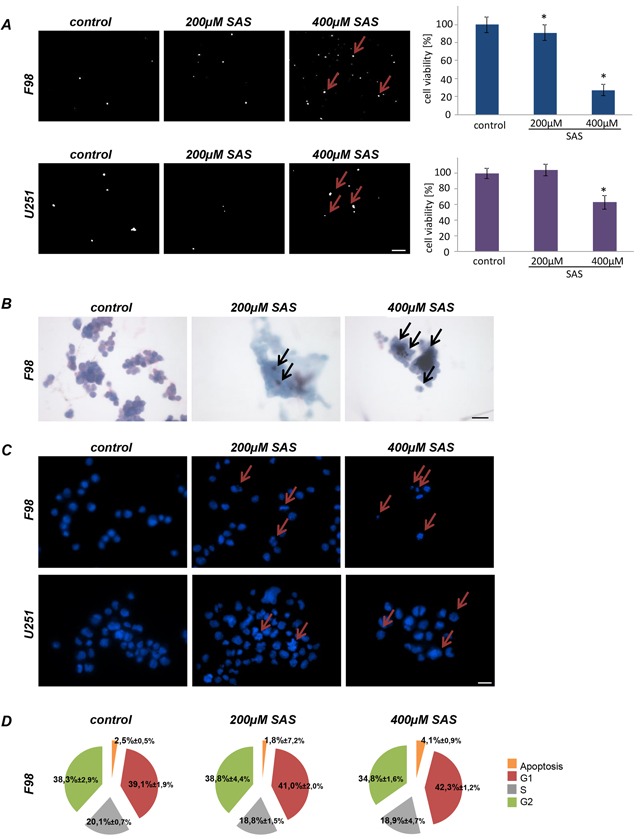
Sulfasalazine induces glioma cell death at high concentrations *in vitro* **A.** Cell death of rat glioma cells (F98) and human glioma cells (U251) were examined after SAS treatment with propidium iodide (PI). Cell viability in F98 was significantly reduced at the indicated SAS concentrations. U251 showed decrease cell viability at 400 μM SAS. Scale bar represents 100 μm. Differences were considered statistically significant with values mean ± SD (n ≥ 3 per group; unpaired two-sided t-test, p < 0.05). **B.** Wright staining for apoptosis detection in rat glioma cells is shown. Glioma cells were treated with SAS. The staining confirmed the identified cell death measurements found with PI staining and the cell viability measurements. Scale bar represents 20 μm. **C.** Hoechst assay was performed to monitor apoptotic morphology in rat (F98) and human glioma cells (U251). Scale bar represents 20 μm. **D.** SAS treated rat glioma cells (F98) were examined for cell cycle status. An increase in apoptosis is visible with higher SAS concentration. Other cell cycle parameters such as G1, S and G2 did not change significantly in comparison to controls.

We investigated the effects of SAS on malignant gliomas. For this we utilized rat and human glioma cell lines (F98, U251; respectively). We tested both glioma cell lines with two different concentrations of SAS for 96 h. Rat glioma cells (F98) showed a significant reduction of cell viability first at a concentration of 200 μM SAS of approximately 12%. At higher level SAS treatment as used in previous reports [14] rat glioma cells responded with morphological challenges and glioma cells started to retract their membrane extensions and rounded up. SAS induced a massive cell death in rat gliomas of over 70% in a dose-dependent manner at 400 μM (Figure 1A). These results were confirmed by morphological analysis for apoptotic characteristics (Figure 1B, 1C). We next investigated the effects of SAS on the human glioma cells U251 (Figure 1A). SAS was effective in impairing cell growth in human glioma cells at a concentration of 400 μM SAS, whereas 200 μM SAS was not toxic. The decreased cell viability counted approximately 35%. Subsequently, we performed cell cycle analyses in glioma cells after SAS treatment (Figure 1D). There was an increase in the apoptotic fraction under SAS application in comparison to controls. Hence, other cell cycle parameters did not change under SAS (Figure 1D).

### Sulfasalazine induces ER stress and ferroptotic cell death without affecting autophagic flux

**Figure 2:**
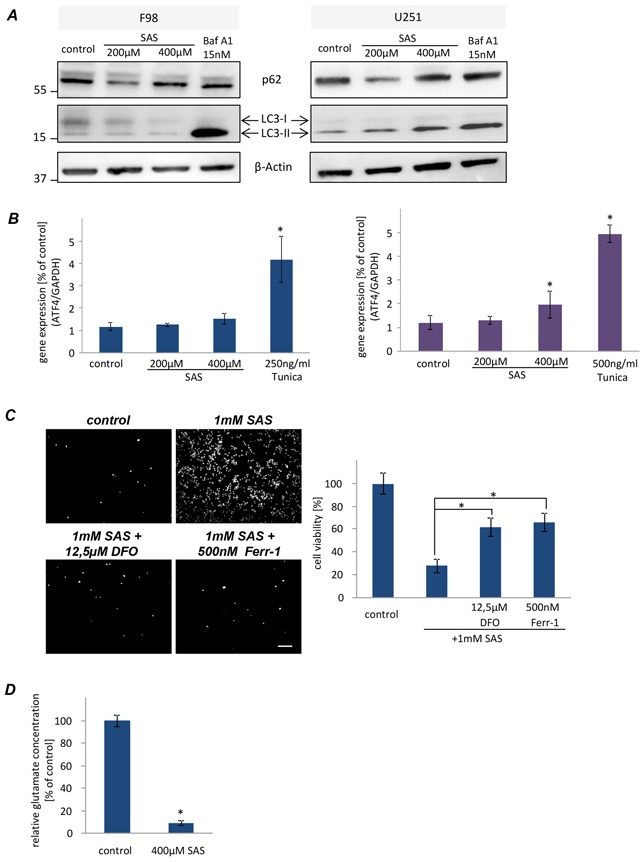
Sulfasalazine induces ferroptotic cell death in glioma cell **A.** Western blot analysis of F98 and U251 glioma cells under SAS treatment for 24 h directed against LC3 and p62 to monitor the autophagic activity. F98 showed no autophagic activity in comparison to U251. **B.** Examination of ER stress response via ATF4 challenges in glioma cells after SAS treatment. F98 displayed no ER stress under treatment in comparison to the ER stress inducer Tunicamycin. U251 showed only at 400μM a slightly higher ATF4 expression. **C.** Monitoring ferroptotic cell death after SAS treatment. 1 mM SAS, ±desferoxamine (DFO) and ferrostatin-1 (Ferr-1) was added to F98 and U251 glioma cells. Scale bar represents 100 μm. SAS induced ferroptosis at very high concentrations. Values: mean ± SD (n ≥ 4 per group; unpaired two-sided t-test, p < 0.05). **D.** SAS inhibits the xCT antiporter activity. Extracellular glutamate release was monitored in untreated glioma cells (controls) and glioma cells after SAS treatment (400 μM). (n ≥ 3 per group; unpaired two-sided t-test, p < 0.05).

To address on the mechanisms of autophagy under SAS treatment, we performed immunoblot analyses using two classical protein markers of autophagosomes LC3 and p62 [15,16]. Specifically, the lipidated form of LC3 (LC3-II) is a structural component of the inner and outer autophagosomal membrane, while p62 accompanies poly–ubiquitinated proteins to the autophagosomes, ultimately becoming a substrate of autophagy itself [17]. Both F98 and U251 were treated with SAS and compared to the autophagic flux inhibitor Bafilomycin A1 (Baf A1) which we used as a modulator known to impair autophagy. As expected, treatment with Baf A1 inhibited autophagy, reflected by accumulation of LC3–II in the cell lysate. However rat glioma cells displayed no LC3–II accumulation, suggesting that SAS did not affect autophagy in this paradigm (Figure 2A). In contrast, in U251 we found a SAS-dependent LC3 – II increase (Figure 2A). Hence, we performed ATF4 expression analyses to examine the ER stress response of glioma cell lines under SAS treatment. We treated rat and human glioma cells with SAS and the ER stress inducer Tunicamycin (Tunica) served as a positive control (Figure 2B). Rat glioma cells revealed a slight but not significant upregulation of ATF4 at 400 μM SAS (Figure 2B). In human U251 gliomas the ATF4 expression was significantly elevated after 400 μM SAS application (Figure 2B). Hence, to investigate whether SAS induces ferroptosis we treated glioma cells with 1 mM SAS for 24 h. SAS showed a significant cell viability reduction with over 70% (Figure 2C). Combined treatment of SAS with desferoxamine (DFO) and ferrostatin-1 (Fer-1), both known iron chelators and inhibitors of ferroptosis, rescued SAS-induced cell death (Figure 2C). We further analyzed whether SAS specifically inhibits the xCT antiporter activity hallmarked by extracellular glutamate release. For that we determined the glutamate secretion into the extracellular space as a direct function of xCT [4]. Here, we found that SAS at high concentrations inhibited specifically the xCT antiporter activity (Figure 2D). Thus, we found evidence that SAS at high concentrations inhibits xCT and induces ferroptotic cell death in glioma cells.

### SAS has minor impact on astrocytes and does not affect neuronal viability

**Figure 3:**
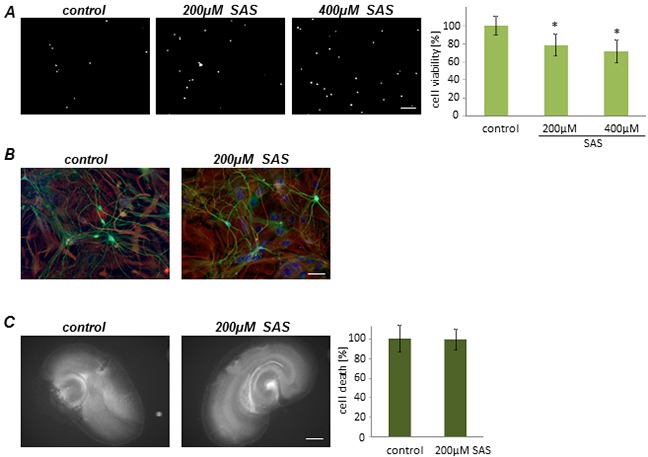
Sulfasalazine impact on primary astrocytes and neurons **A.** Primary astrocytes were treated with SAS. Cell death and viability measurements showed a viability reduction after SAS treatment. Scale bar represents 100 μm. Differences were considered statistically significant with values mean ± SD (n ≥ 4 per group; unpaired two-sided t-test, p < 0.05). **B.** Neurons were treated with SAS and further stained for the neuronal marker beta-III- tubulin (green). There are no toxic effects visible in neurons. Scale bar represents 50 μm. **C.** SAS treatment shows no neuronal damage in native brain tissue. After 5 days in culture cell death was monitored (white signal). Scale bar represents 1 mm. Values are given as mean ± SD and differences are considered statistically significant with *P < 0.05 (unpaired two- sided t-test, n ≥ 9 per group).

This data led to the question of whether SAS has general toxic effects on proliferating cells or whether the observed effects are specific for glioma cells. To tackle this we facilitated primary rat astrocytes and treated these cells with SAS. Monitoring of cell death revealed that SAS treatment is slightly toxic towards astrocytes (Figure 3A). At 200 μM SAS induced diminished cell viability to 80% compared to controls and at 400 μM SAS reduced cell viability to 30% (Figure 3A). We expanded this study and investigated the effects of SAS on neurons (Figure 3B). SAS treated neurons showed no visible toxic effects (Figure 3B). We further examined the impact of SAS treatment on neuronal integrity and survival in native organotypic brain slices (Figure 3C). With this *ex vivo* model it is possible to test pharmacological substances in a real time mode [18], [19]. Brain sections were prepared and slices were cultured on permeable PET membranes bathed in culture medium. Treatment with SAS was performed and cell death was assessed with PI after five days in culture. These experiments showed that SAS was not affecting brain cell viability compared to controls (Figure 3C).

### SAS does not affect tumor growth progression *ex vivo*

**Figure 4:**
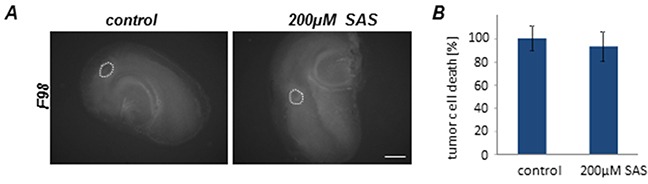
Sulfasalazine treatment is not gliomatoxic within the brain tumor microenvironment Organotypic brain slices (ex vivo) with glioma cell implantation (VOGiM assay) were cultured in the presence of SAS (at 200 μM). **A.** SAS treatment induces no tumor cell death in glioma-implanted brain slices. F98-GFP expressing glioma cells were implanted in brain slices and the size of the tumor bulk was documented after 5 days by fluorescence imaging. Scale bar represents 1 mm. **B.** Tumor bulks of the SAS group and the control group were compared quantitatively. There was no significant difference between these two groups. Differences were considered statistically significant with values mean ± SD (*P < 0.05, unpaired two-sided t-test, n ≥ 11 per group).

Next, we implanted F98 GFP-expressing glioma cells into normal brain tissue slices and used the *ex vivo* model for monitoring tumor growth under organotypic microenvironmental conditions (Figure 4A). Tumor growth was assessed over a course of 5 days. There was no visible and quantitative difference in untreated tumor-implanted control slices in comparison to SAS-treated tumor implanted brain sections (Figure 4A, 4B).

### Impact of combined SAS–Temozolomide application on gliomas

**Figure 5:**
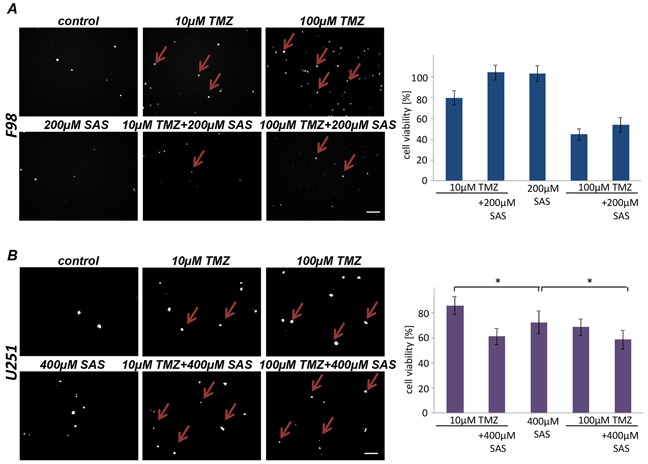
SAS effects in combination with Temozolomide **A.** Cell death and cell viability of rat glioma cells were measured after the treatment with SAS and TMZ itself and their combination. Cell death was determined with propidium iodide (PI). No significant additive effect of SAS and TMZ exists in F98 glioma cells. **B.** Human glioma cell line U251 were treated with a SAS and TMZ and their combination; cell survival (PI) and cell viability was monitored. SAS showed a significant additive effect in combination with TMZ. Differences were considered statistically significant with values mean ± SD (n ≥ 3 per group; unpaired two-sided t-test, p < 0.05).

We next investigated whether SAS can improve the efficacy of the standard cancer chemotherapeutic drug temozolomide. Temozolomide (TMZ) is an alkylating agent which is routinely used in the clinical management of glioblastoma (GBM) patients. We utilized rat and human glioma cell lines and applied SAS and TMZ alone and combination in this setting (Figure 5). Interestingly, SAS did not show any multiplying effect when combined with 10 μM TMZ on rodent gliomas (Figure 5A). This was also the case when TMZ was raised up to 100 μM (Figure 5A). However, combined SAS and TMZ treatment in human U251 glioma cells appeared to be more potent compared to single TMZ application (Figure 5B). A significant cell death for the combination was visible compared to single application of TMZ or SAS alone (Figure 5B).

### SAS alleviates tumor-related brain edema *in vivo*

**Figure 6:**
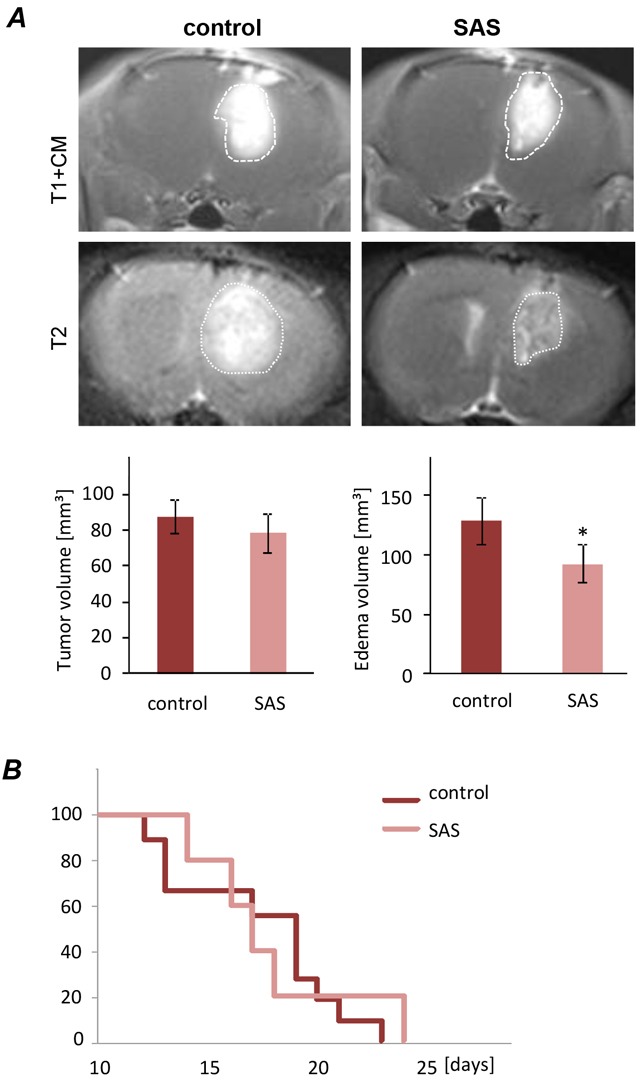
Sulfasalazine treatment alleviates tumor-induced peritumoral edema *in vivo* **A.** Representative MRI images of untreated (control) and SAS treated gliomas at day 10 after tumor implantation. The tumor bulk representing the tumor zone I is marked with dashed white lines and was visualized after application of intraperitoneal contrast agent and subsequent T1-weighted imaging (T1+CM). Bottom images reveal corresponding perifocal edema in T2-weighted scans. Quantification of T1- and T2-weighted MRI scans of 9 control and 5 SAS-treated tumor-implanted rats. Tumor volume was quantified from T1 - weighted MRI images. The edema zone was quantified by subtraction of T2 versus T1-weighted MR volume. T1-weighted Tumor volume corresponding to tumor zone I (TZI) showed no significant difference between SAS-treated rats and the untreated control group. The edema zone is significantly smaller in the SAS-treated group compared to the control group. Statistical significance was calculated with Student's t-test (mean ± SD; n ≥ 5 per group; unpaired two-sided t-test, p < 0.05). **B.** Animals were clinically assessed on a daily basis according to their neurological status (grade 0: normal; grade 1: tail weakness or tail paralysis; grade 2: hind leg paraparesis or hemiparesis; grade 3: hind leg paralysis or hemiparalysis; grade 4: complete paralysis (tetraplegia), moribund stage or death). Therefore the onsets of neurological symptoms were measured. There is no statistical difference according to the development of neurological deficits between animals treated with SAS and the untreated control.

Finally, we investigated whether SAS in gliomas has any effects *in vivo*. For this we orthotopically implanted syngeneic glioma cells into rat brain. The tumor was monitored by magnetic resonance imaging (MRI) scans at 10 days after implantation (Figure 6A). Primary tumor volume corresponding to the tumor zone I (TZ I), as assessed by T1-weighted images at 3 Tesla MRI after paramagnetic contrast administration revealed no differences in rats treated with SAS versus untreated controls. However, to unmask the proper expansion of tumor lesions (meaning tumor volume and perifocal brain edema [4], [20], we acquired T2-weighted scans depicting perifocal edema as high intensity areas surrounding the contrast-enhanced tumor part referring to T1–weighted imaging. In these scans, rats treated with SAS showed significantly alleviated perifocal edema compared to untreated tumor controls. Conversely, rats which received SAS treatment showed a slightly delayed onset of neurological deficits compared to controls (Figure 6B). These data indicate that the perifocal edema reflects the tumor microenvironment and has significant impact on the tumor malignancy. However, evaluation of the Kaplan Meier survival analysis showed no statistical significant differences between SAS-treated rat and their untreated controls (Figure 6B).

## DISCUSSION

In this study we investigated the efficacy and adverse effects of the FDA-approved agent SAS. The rationale for this came from previous reports indicating that SAS is a potent pharmacological agent inhibiting the glutamate antiporter xCT [12], [21]. Numerous studies demonstrated that glioma cells secrete high levels of the neurotransmitter glutamate, resulting in neuronal cell death [4], [22], [23], [24] and brain edema [4] via excitatory activation of neuronal glutamate receptors. Glutamate receptor antagonists block neuronal degeneration in the tumor vicinity and lessen glioma growth *in vivo*, suggesting that neurodegeneration is a prerequisite for rapid glioma progression [25]. In addition, the antiporter system X_c_^−^, mediating glutamate release in exchange for cystine, is abundantly expressed in glioblastoma specimens and cell lines [4], [24], [26], [27]. Reduced EAAT1 and EAAT2 expression and concurrently abundant system X_c_^−^ activity (old nomenclature of xCT activity) results in a net balance shift towards glutamate release, thus promoting glioma progression. Moreover, inhibition of glutamate release via system X_c_^−^, consisting of xCT (SLC7A11) and CD98 (SLC3A2, 4F2HC), profoundly decelerates the glioma phenotype *in vivo* [4], [12], [26], and in addition mitigates tumor-induced brain swelling [4] and tumor-induced seizures [13]. Even though the glioma-promoting properties of glutamate release by glioma cells appear to be without doubt, the underlying mechanisms are still poorly understood. Besides neurotoxic effects, some studies assume that extracellular glutamate promotes glioma proliferation in an autocrine or paracrine fashion via activation of ionotropic glutamate receptors on gliomas, especially of the AMPA subtype [28]. Thus xCT is at the center stage for glutathione dependent redox regulation and glutamate homeostasis. Sufasalazine has been found to reduce epileptic activity at high concentrations (SAS at 250 μM) in glioma-bearing mice, implicating extracellular glutamate elevation in the generation of tumor-associated epileptic events [13]. Moreover, there are indications that xCT is implicated in tumor-associated epileptic events in humans [14]. In fact, SAS is a FDA-approved drug with well-know and tolerable side effects commonly used in Crohn's disease. We tested SAS in gliomas and found chemotherapeutic effects solely at concentrations from 200 μM upwards. These findings have been also confirmed *in vivo*. There, SAS was not effective in reducing tumor growth and primary tumor volume. However, we found that SAS was efficient in alleviating tumor-induced brain swelling as revealed by T2-weighted MRi scans, indicating the impact of xCT on the tumor microenvironment. These findings are in congruence with *in vivo* studies with the experimental xCT inhibitor S-4CPG [4].

Clinically, SAS has been tested in two independent phase I clinical trials, one at the University of Alabama at Birmingham (USA) and one at the University of Liège (Belgium). In an open phase I study at Birmingham (USA) glioma patients were treated with SAS and monitored by MRi imaging. Although this trial included five patients which responded well to orally applied SAS, this study has not been continued for funding issues (H. Sontheimer, personal communication). The second prospective clinical trial has been enrolled in Belgium (EU) including a small cohort of patients suffering recurrent or progressive WHO grad III-IV gliomas after surgery [29]. Specifically, 8 patients were diagnosed with recurrent glioblastomas multiforme and two patients were diagnosed with progressive anaplastic astrocytoma, and overall median Karnofsky score was 50, indicating already the low performance and inability of patients in daily life and requirement for frequent medical care. Patients received continuously orally applied SAS at a concentration between 1.5 to 6 grams. However, before a greater study population was included this clinical trial was terminated in first line due to a lack of clinical response and in addition due to the high frequent adverse effects (grade I-III in all 10 patients) [30]. Although solely a small patients' cohort has been tested so far, efficacy of SAS at least in recurrent gliomas and anaplastic astrocytoma is not evident.

Does this mean SAS is not effective at all in gliomas? And how can this are explained out of *in vitro* and animal models? Although one can argue that this prospective trial falls short due to too restrictive inclusion criteria and overall weak patients conditions, the problem may is two-sided. Since the motivation for using SAS in patients suffering from gliomas comes from data that SAS inhibits NFκB, we revisited data on SAS processing and its molecular action. Initial studies demonstrating that SAS inhibits NFκB activation already indicated that in fact SAS acts on NFκB whereas is breakdown products and metabolites 5-ASA and sulfapyridine are not effective [10]. Critical evaluations of these data further reveal that SAS is hence solely a weak NFκB inhibitor with an EC_50_ of approx. 0.6 mM in NFκB luciferase activity assays [10], [11].

A novel finding is that SAS can potentiate the standard chemotherapeutic TMZ in human gliomas. This finding is of clinical relevance and in addition with reports of SAS as a sensitizer for radiotherapy [31] could implement this xCT inhibitor as a new modality in glioblastoma therapy. But in comparison to TMZ, SAS–mediated effects on glioma cell survival did not appear to rely on significant alterations in the mechanism of autophagy [32]. Instead, SAS can induce ferroptosis in glioma cells. Thus TMZ and SAS act on different cell death pathways which can in principle potentiate efficacy. However, so far we found clear evidence for tumor microenvironment normalizing activity of SAS *in vivo*.

The xCT inhibitory dosages we observed was at 200 μM SAS onwards which is in line with previous reports using effective concentrations of 250 μM SAS [13]. In fact 250 μM SAS means 7 g SAS per day at body weight of 70 kg. These dosages are approximately double of what is routinely used in Crohn's disease. Thus, more potent SAS compounds are required to target the glutamate antiporter xCT in clinical conditions.

Another line would be the multimodal use of SAS for supporting already established standard chemotherapeutic agents for diminishing the dreaded brain swelling. However, to further route this pathway more potent SAS analogues are needed to target the glutamate antiporter xCT.

## MATERIALS AND METHODS

### Cell culture

Rodent glioma cell line F98 was obtained from ATCC/LGC-2397 (Germany). Primary rat astrocytes were prepared from up to one month old Wistar rats. All cells were cultured under standard humidified conditions (37°C, 5% CO_2_) with Dulbecco's Modified Eagle Medium (DMEM; Biochrom, Berlin, Germany) supplemented with 10% fetale bovine serum (Biochrom, Berlin, Germany), 1% Penicillin/Streptomycin (Biochrom, Berlin, Germany) and 1% Glutamax (Gibco/Invitrogen, California, USA). Cells were passaged at approx. 80% confluence. Cells were trypsinized after PBS wash step. After centrifugation (900 rpm for 5 min) cells were plated out in culture flask.

### Chemicals

Sulfasalazine (SAS) were purchased from Sigma-Aldrich (Taufkirchen, Germany). Sulfasalazine was dissolved in 400 mM ammonium hydroxide under sterile conditions to concentration of 200 mM. Bafilomycin A1 and Tunicamycin were purchased from AppliChem (Darmstadt, Germany). Bafilomycin A1 was diluted with DMSO to a stock of 200μM and Tunicamycin in DMSO to 2,5 mg/ml. Desferoxamine (DFO) and Ferrostatin-1 (Ferr-1) were purchased from Sigma-Aldrich (Taufkirchen, Germany). Desferoxamine was dissolved in water under sterile conditions to a concentration of 50 mM. Ferr-1 was prepared in 50% DMSO/water under sterile conditions to a concentration of 50 mM.

### Cell viability analysis and toxicity assays

The cell viability assay was performed using 3(4,5 dimethylthiazol) - 2,5 diphenyltetra-zolium (MTT) assay according to [33] the cell viability was measured. 3000 cells/well were plated in 96 well - plates one hours prior to the drug treatment. On the fourth day cells were incubated with MTT solution (Roth, Karlsruhe, Germany) (5 mg/ml) for 4 h at 37°C, 5% CO_2_. The lysis of the cells occurred with 100 μl isopropanol + 0.1 N HCl. The optical density of each well was determined using the microplate reader Tecan Infinite F50 (Crailsheim, Germany) set to 550 nm (wavelength correction set to 690 nm) using Magellan software. Plates were normally read within 1h of adding after lysis. Control was cells without drugs. The viability of the cells was expressed as the percentage of control. Assays were performed on at least three independent experiments.

### Cell death assay and apoptosis analysis

For the cell death assay cells were seeded and treated the same way as the MTT assay. Cells were incubated with propidium iodide staining (PI) purchased from Molecular Probes (Invitrogen, Darmstadt, Germany). Cells were stained 20 min [1 μg/ml]. Apoptosis analysis was conducted with Wright and Hoechst staining. For the Wright staining sells were seeded 80000 cells/well in 6 well-plates five hours prior to the drug treatment. Cell death assay was performed on the fourth day. Cells were washed with PBS and then fixed and subjected to the Wright staining according to the manufacturer's instructions (Sigma-Aldrich, Taufkirchen, Germany). After staining, cells were dried and embedded. The Hoechst staining was performed with modifications as described previously [34]. Thus, cells plated at 25.000 cells/well in a 12 well-plate and treated after 1h with the drug. Following incubated for 4 days. Supernatant were collected, samples were washed with PBS (saved) and trypsinized. The pooled samples were fixed with 4% PFA on ice for 10 min, and then spun down at 1500 rpm for 5 min. Cells were stained with HOECHST 33258 (Invitrogen, Darmstadt, Germany) [1 μg/ml] for 20 min in darkness. The suspension was added to an objective glass and mounted on cover slips. Afterwards morphological features of apoptotic cells were observed under an Olympus x71 and images were taken with cell-F software (Olympus, Tokyo, Japan). The same equipment was used for the cell death assay. Images for the Hoechst staining were taken by an Axio Observer with the Zen Software (Zeiss, Oberkochen, Germany).

### Primary neuronal and astrocytes cultures

Hippocampal neuronal cultures were prepared from one to four days old Wistar rats (Charles River, USA). Briefly, newborn rats were sacrificed by. Hippocampi were removed from the brain and transferred into ice cold Hank's salt solution, and the dentate gyrus was cut away. After digestion with trypsin (5 mg ml^−1^) cells were triturated mechanically and plated in MEM medium, supplemented with 10% fetal calf serum and 2% B27 Supplement (all from Invitrogen, Taufkirchen). In brief, the culture medium was removed and replaced with Neurobasal A (Invitrogen, Taufkirchen). The culture was provided by Carmen Christoph (Department of Psychiatry, Erlangen University Hospital). Neurons were stained with beta-III-tubulin (1:500, Promega, Madison, Wisconsin, USA), astrocytes with GFAP (1:500, Dako, Glostrup, Denmark) and counterstained with Hoechst 33258 (1:10000, Life Technologies, Darmstadt, Germany). Images were taken by an Axio Observer with the Zen Software (Zeiss, Oberkochen, Germany).

### Cell cycle analysis

80000 cells/well were seeded in 6 well – plates and treated five hours later with drugs. Cell cycle analysis was performed on the fourth day with Flow Cytometer BD FACSCanto II (BD Bioscience, Heidelberg, Germany). Cells and media supernatant were collected. The pellet were washed with PBS and afterwards resuspend in PI-Hypotonic lysis buffer (PI - LB: 0,1 % sodium citrate, 0,1% Triton X - 100, 100 μg/ml RNAse). Cell cycle analyses were performed within 2 h after adding 7 - AAD (7 - aminoactinomycin D, Molecular Probes, Invitrogen, Darmstadt, Germany). Analyses were carried out with Flowing Software 2 (Turku Center for Biotecnology, University Turku, Finland).

### Organotypic brain slice cultures (OGIM) and native brain slice cultures

Brain slice cultures were conducted with five days old Wistar rats (Charles River, Boston, MA, USA). Brains were prepared and maintained as previously described [18]. Animals were sacrificed; brains were removed and kept under ice - cold conditions after the frontal lobes and cerebellum were dissected of the hemispheres. The brain was cut into 350 μm thick horizontal slices using a vibratome (Leica VT 1000S, Bensheim, Germany). The brain slices were transferred ontoThinCert™ cell cultureinserts (GreinerBioOne, Frickenhausen, Germany; pore size 0.4 μm) and following cultured under humidified atmosphere (35°C, 5% CO2) in 6-well culture dishes (GreinerBioOne, Frickenhausen, Germany) containing 1.4 ml culture medium (MEM-HBSS, 2:1, 25% horse serum, 2% L-glutamine, 2.64 mg/ml glucose, 100 U/ml penicillin, 0.1 mg/ml streptomycin, 10 μg/ml insulin–transferrin-sodium selenite supplement and 0.8 μg/ml vitamin C). The medium was changed on the first day after preparation and from that time on every second day. The drug treatment started the day after the preparation and correlated with the medium change. Stably GFP--transfected F98 glioma cells [10.000 cells] (p–EGFP-N1 from BD Biosciences Clontech, Heidelberg, Germany) were implanted within a total volume of 0.1 μl medium into the entorhinal cortex (layer II and III) one day after slice preparation. Glioma growth and cell death were evaluated one day after implantation and then every second day using the microscope Olympus ix71. Slice cultures were incubated with 1 μg/ml PI for 20 min followed by complete medium exchange in order to visualize irreversibly damaged cells and apoptosis. Slices were fixed with Immunfixative (PFA, Picric acid) after 5 days.

### RNA isolation and qRT - PCR experiments

F98 (400.000 cell/well) and U251 (250.000 cell/well) were seeded in a 6–well plate two hours before drug treatment, incubation of 24 h followed. Just Tunicamycin on F98 cells were incubated only for 6 h. Cells were washed with PBS and Trizol (Peqlab, Erlangen, Germany) was added. Cells were collected RNA was isolated according to the manufacture's protocol. RNA concentration was quantified by NanoVue™ Plus Spectrophotometer (GE Healthcare, UK). cDNA was synthesized with 1 μg of total RNA using DyNAmo cDNA Synthesis Kit (Biozym, Hessisch Oldendorf, Germany) according to the manufacturer's protocol. Real-time (DyNAmo ColorFlash SYBR Green qPCR Kit) PCR was performed in a LightCycler® 480 (Roche Applied Sciences) according to the manufacturer's protocol (Biozym, Hessisch Oldendorf, Germany). The oligos used in this study are: ATF4 forward primer: GGTTCTCCAGCGACAAGG; ATF4 reverse primer: TCTCCAACATCCAATCTGTCC. GAPDH forward primer: TGCACCACCAACTGCTTAGC; GAPDH reverse primer: GGCATGGACTGTGGTCATGA. Real time cycling parameters: Initial activation step (95°C, 15 min), cycling step (denaturation 94°C, 15 s; annealing 60°C, 30 s; and extension 72°C, 30 s X 45 cycles), followed by a melting curve analysis to confirm specificity of the PCR. All samples were assessed in relation to the levels of GAPDH expression as an internal control. Q-PCR data were assessed and reported according to the ΔΔCt method. Data from at least five determinations (means ± SD) are expressed as relative expression level.

### Protein isolation and immunoblotting

F98 (400.000 cell/well) and U251 (250.000 cell/well) were seeded in a 6–well plate two hours before drug treatment, following a 24 h incubation. For protein extraction samples were lysed with NP 40 buffer containing a protease inhibitor cocktail (Roche, Basel, Switzerland). Afterwards homogenization by ultrasound (Bandelin Sonoplus, at 67 %) and incubated on ice for 10 min. Samples were centrifuged at 8000 rpm for 10 min at 4 C. Concentration was measured with NanoVue™ Plus Spectrophotometer (GE Healthcare, UK). Samples were mixed with loading buffer (4x) and Reducing agent (10x) (Invitrogen, California, USA) and boiled at 90°C for 5 min. 10μl of protein sample were loaded on 4 - 12 % SDS-NuPage Gel (Invitrogen, CA, USA). Electrophoresis was performed with MOPS–buffer (Invitrogen, CA, USA), transferred on PVDF membranes (Roth, Karlsruhe, Germany) with NuPage Transfer buffer (Invitrogen, CA, USA). Membranes were blocked in PBS containing 2 % Magic block and 10 % Top block (Lubio science, Lucern, Switzerland) for 1 h before further processed. Antibodies were incubated overnight at 4°C in roller tubes, followed by HRP-secondary antibodies incubated at room temperature for 1 h. Detection was performed with ECL plus kit (GE-healthcare, Solingen, Germany). Primary Antibodies: p62 (1:1000, MBL, Massachusetts, USA), LC3 (1:200, Novus Biologicals, Colorado, USA) and β-Actin (1:1000, Santa Cruz, Texas, USA). The blot was performed three times with three independent repetitions of each group of proteins.

### Amino acid profiling of glioma conditioned medium

Cells were seeded in 12-well plates at a density of 200.000 cells/well in DMEM supplemented with 10% FBS, 1% P/S and 1% Glutamax. After incubation overnight the cells were 80 % confluent. The medium was changed to DMEM without supplements and drugs were added. After incubating for another 12 hours, medium was collected and measurement was performed by HPLC. Amino acids were analysed by ion-exchange chromatography and post-column ninhydrin derivatization technique using a fully automated amino acids analyzer (Biochrom 30+, Laborservice Onken, Gründau, Germany). For the amino acid analysis, 100 μL of sample was deproteinised with 100 μL of 10% sulphosalicylic acids. 20 μL of this supernatant was then loaded by the autosampler into a cation-exchange resin-filled column. Three independent experiments were performed.

### *In vivo* study

Animal experiments were performed Frankonian State approval and in congruence with the European Union guidelines governing the use of laboratory animals (no.5425310806). All efforts were made to reduce animal numbers and pain suffering. Male Fisher rats (Charles River, Boston, MA, USA) were deeply anesthetized by intraperitoneal injection of ketamine (Pfizer, Germany)/xylazin (Bayer Healthcare, Germany) (2:1) before fixing them in a stereotactic frame (David Kopf Instruments, Bilaney Consultants). Stably GFP-transfected F98 rat glioma cells were stereotactically implanted in a volume of 4 μl (100.000 cells) with a Hamilton syringe (VWR, USA) into the right frontal lobe of the animals (2 mm lateral to bregma, 4 mm depth from dura). Drug treatment started the second day after implantation, thereafter every second day SAS was applied intraperitoneally. SAS dissolved in sterile water (used as vehicle) was administered through intraperitoneal injection (200 μl each time) at 50 mg/kg. Tumor implant was monitored 10 days after implantation using a clinical 3 Tesla MRI scanner (MAGNETOM TimTrio, Siemens Healthcare, Siemens AG, Erlangen, Germany). A modified neurological deficit assessment was documented according to four clinical scales [4]. In brief, rats were clinically checked every day and evaluated according to neurological status (grade 0: normal; grade 1: tail weakness or tail paralysis; grade 2: hind leg paraparesis or hemiparesis; grade 3: hind leg paralysis or hemiparalysis; grade 4: complete paralysis (tetraplegia), moribund stage or death). Rats were sacrificed at grade 4. Grade 1 was defined to be the onset of neurological deficit.

### MRI determination of brain edema determination

MR imaging was performed on a clinical 3 Tesla MR scanner with a 40 mm-diameter, small field-of-view orbita surface coil as receiver. Scout images and a 3DCISS sequence (repetition time=9 ms, echo time=5 ms reconstructions with a slice thickness of 2 mm) were obtained in coronal, axial, and transverse planes to position the slices accurately. Ten coronal T1- and T2-weighted slices, each with 2 mm thickness and 0.2 mm separation (inter-slice gap) were then positioned on the transverse scout images to cover the whole tumor. T1-weighted images were acquired with a 384×307 matrix, field-of-view=70×70mm, repetition time=507 ms, echo time=17 ms, and a total scan time of 3 min 42 s. For contrast enhanced images, each animal received 0.1 ml per kg body weight of contrast agent (Gadovist 1.0, Bayer Pharma, Leverkusen, Germany) intravenously 10 min prior to the acquisition of T1-weighted sequences. T2-weighted images were acquired with a 320×256 matrix, field-of-view=91×91 mm, repetition time=4500 ms, echo time=158 ms, and a total scan time of 6 min 12 s. Imaging analysis was performed for each rat using Osirix (GNU General Public License build-in image processing software) to outline tumor volume on the T1-weighted contrast-enhanced images. Total tumor volume was calculated as the summed area on all slices, multiplied by the slice separation and compared to histology-derived tumor volume. Additionally, edema volumes were measured by subtracting the contrast-enhanced tumor volumes referring to T1-weighted imaging from the complete hyperintense tumor volumes referring to T2-weighted-imaging.

### Statistical analysis

Analysis was performed using unpaired Student's t-test (MS Excel). Data from cell viability analysis and toxicity assays were obtained from at least four independent experiments. The level of significance was set at *p < 0.05. Error bars represent ± SD.
